# A Novel Physically Guided Data Fusion Prediction Model for Micro-EDM Drilling

**DOI:** 10.3390/ma16237454

**Published:** 2023-11-30

**Authors:** Chen Cheng, Beiying Liu, Jinxin Cheng, Xiao Xiong

**Affiliations:** 1 School of Mechanical Engineering, University of Science and Technology Beijing, Beijing 100083, China; m202120518@xs.ustb.edu.cn (C.C.); chengjinxin@ustb.edu.cn (J.C.); 2 School of Engineering, Hong Kong University of Science and Technology, Hong Kong, China; xiongxiao917@gmail.com

**Keywords:** EDM, MRR, modeling, shape, BP-ANN, GA

## Abstract

Accurate prediction of Electro-Discharge Machining (EDM) results is crucial for industrial applications, aiming to achieve high-performance and cost-efficient machining. However, both the current physical model and the standard Artificial Neural Network (ANN) model exhibit inherent limitations, failing to fully meet the accurate requirements for predicting EDM machining results. In addition, Micro-EDM Drilling can lead to the distortion of the macroscopic shape of machining pits under different input conditions, rendering the use of only the volume of machining pits as the evaluation index insufficient to express the complete morphological information. In this study, we propose a novel hybrid prediction model that combines the strengths of both physical and data-driven models to simultaneously predict Material Removal Rate (MRR) and shape parameters. Our experiment demonstrates that the hybrid model achieves a maximum prediction error of 4.92% for MRR and 5.28% for shape parameters, showcasing excellent prediction accuracy and stability compared to the physical model and the standard ANN model.

## 1. Introduction

EDM is a non-traditional, non-contact machining method that utilizes electrical energy to generate thermal energy, leading to thermal corrosion on the surface of the material being machined and causing material removal [1]. Traditional machining methods often face challenges when working with hard materials, and the resulting machined parts may have residual stresses, which can negatively impact their performance and behavior. Elimination of these residual stresses requires additional processes, consuming time and economic resources [2]. The unique machining mechanism of EDM enables it to overcome the limitations of conventional cutting processes [3]. However, MRR and machining quality of EDM based on the principle of thermal corrosion are typically lower than those achieved by conventional cutting methods [4]. Therefore, achieving accurate prediction of the EDM machining process and its outcomes has become a significant research focus. This endeavor aims to enhance the machining efficiency and precision of EDM operations.

A part of scholars is devoted to the optimization of process parameters for EDM, investigating the contribution of each parameter and its interactions in EDM, and finding the optimal combination of parameters to achieve the enhancement of MRR [5]. Modeling the EDM process serves as a prerequisite for optimization efforts, where a well-constructed model can express the influence of each input parameter and its interaction with the output results. Presently, two primary categories of models are prevalent: physical models based on the fundamental principles of the EDM process and data-driven models based on statistical analysis. Physical models are grounded in the processing mechanisms, quantifying the effects of specific reactions and processes in EDM to calculate the processing results. These models often involve the use of computer-aided calculations and finite element analysis (FEA) to achieve accurate predictions [6,7]. Through the efforts of numerous studies, there developed a profound understanding of the various input parameters in EDM and the specific phenomena occurring during the process. This resulted in physical models with high prediction accuracy [8,9]. For instance, the latest physical prediction model proposed by Sahoo reportedly exhibits a controlled error of only 13% within a suitable range [10]. A common approach for numerical predictions in EDM involves the utilization of numerical prediction models based on two main methodologies: orthogonal experiments and response surface analysis (RSM). These techniques are employed to optimize process parameters and enhance the EDM performance for specific applications [11,12,13]. In addition to traditional numerical methods, some scholars have adopted emerging AI methodologies, particularly by building standard ANN models. These ANN models are trained using a significant amount of data to establish mappings between input parameters and output results [14,15].

Current research demonstrates that AI techniques hold significant promise in predicting EDM machining results. Ashutosh Kumar Singh [16] investigated the influence of various input parameters, such as peak current, open circuit voltage, and pulse parameters, on MRR and Tool Wear Rate during the EDM of Al7075. They developed an ANN prediction model with a mean absolute error (MAE) of 3.99%. However, this model exhibited technical limitations, leading to instability in prediction errors. It demonstrated minimal prediction errors for some data points but large prediction errors for others. M. Arunadevi [17] compared the prediction performance of an ANN model with linear regression prediction. Based on experimental results, they concluded that the ANN model’s predictions were more stable than those of the linear regression model. Additionally, M. Arunadevi combined ANN with Pareto optimization to find optimal prediction results that satisfy both MRR and Surface Roughness metrics. Mariangela Quarto [18] developed a Particle Swarm Optimization (PSO) algorithm-based ANN model and compared it with the FEA model. The results showed that the PSO-ANN approach exhibited smaller prediction errors in terms of MRR, while machining time was a better predictor for both models. From a modeling perspective, the PSO-ANN model was found to be more applicable than the FEA model, requiring less time and being easier to implement. Moreover, Machno [19] and Mondal [20] compared the RSM model with the ANN model under different machining conditions. They both found that the ANN model outperformed the RSM model, showing smaller prediction errors.

Most of the current studies employing data-driven modeling for predicting EDM results have predominantly utilized the standard ANN model to predict the MRR of the EDM process, with some incorporating the prediction of post-processing Surface Roughness. However, in the specific prediction process, the dominant model consists of multiple input parameters predicting one output result. One of the challenges in applying the ANN algorithm in practice is determining a reasonable network structure. Table 1 displays the network structures used in current studies on ANN prediction of EDM results, and the majority of them utilize a three-layer network, which consists of one hidden layer. This choice is rooted in Robert Hecht-Nielsen’s proof that any continuous function in a closed interval can be approximated by an ANN network with one hidden layer [21]. Therefore, a three-layer ANN network is considered sufficient for handling multiple inputs and multiple outputs. The number of units in the hidden layer significantly impacts prediction results. If the number of units is too small, the network may not capture enough information to effectively solve the problem. Conversely, if the number of units is too large, it can increase the time required for training and may lead to the overfitting problem, resulting in increased test error. In summary, compared to current physical prediction models, the ANN prediction model offers more convenience and simplicity, with higher prediction accuracy. It presents a promising approach for accurately predicting EDM results.

However, it is worth noting that despite the advancements in both physical and data-driven models in current research, most of these models still suffer from large prediction error. For example, the physical model proposed by Sahoo includes numerous simplifications, leading to a maximum prediction error of 13%. The inherent error in the physical models is attributed to their inadequacies and limitations. The ANN model developed by Ashutosh has an average error of 3.99%, but for specific input values, its prediction error exceeds 20%, indicating that the ANN model lacks a comprehensive understanding of the intricate relationship between each input and output in the EDM process.

The current research predominantly focuses on predicting the MRR of EDM. While MRR is a vital performance evaluation metric for EDM, it may not be sufficient in practical manufacturing scenarios, especially in precision machining applications like Micro-EDM Drilling. In such cases, the accuracy of the machining results in terms of macroscopic morphology becomes crucial. Maintaining the precision of initial machining results is critical to minimizing the economic and time costs associated with subsequent finishing operations. Consequently, accurate prediction of features strongly correlated with morphological parameters in EDM microvia machining has become a crucial research direction with promising applications in the manufacturing industry. By conducting in-depth research and accurate prediction of features related to morphological parameters in EDM microvia machining, more precise parameter settings and control strategies can be provided for the EDM microvia machining process. This aids manufacturers in cost reduction, productivity improvement, and ensuring that product quality meets requirements, ultimately providing practical solutions to real-world manufacturing needs. Implementing such approaches can offer manufacturers improved economic benefits, competitive advantages, enhanced product quality, and increased customer satisfaction. However, it is essential to acknowledge that due to the strong randomness inherent in EDM machining as a combination of multiple physical phenomena and the complexity of the machining results after subjecting a workpiece to hot corrosion, the current prediction models still face challenges in accurately predicting the shape accuracy of the machining results.

In light of the existing challenges, both the inherent defects of current models and the limitations in predicting sufficient parameters, the industry’s pursuit of enhanced machining efficiency and accuracy in EDM necessitates the development of a reliable model capable of handling complex machining scenarios and recognizing interrelationships between machining results. To address these issues and achieve accurate prediction in EDM microvia machining, this study undertook the following efforts:

1. Investigated the dynamic effects of changes in input parameters on the shape parameters of machining results in EDM microvia machining. The study explored the correlation roles between the volume, surface area, and depth of machined pits in the machining results.
2. Developed a hybrid model that combines physical and data-driven models using the BP-ANN algorithm and genetic algorithm for EDM microvia machining. This hybrid model was designed to predict the machining rate of EDM and three morphological parameters with strong correlations in the machining results. The study also evaluated statistical metrics, such as average prediction error and maximum prediction error, to assess the model’s performance.
3. Investigated and compared the prediction results of the latest physical model, the standard ANN model, and the new hybrid model proposed in this study using the same dataset. The study examined the statistical metrics of each model’s prediction results and pointed out the shortcomings of the physical prediction model and the standard ANN prediction model, as well as the outstanding advantages of the new hybrid prediction model.

## 2. Materials and Methods

### 2.1. Experimental Details

**Figure 1:**
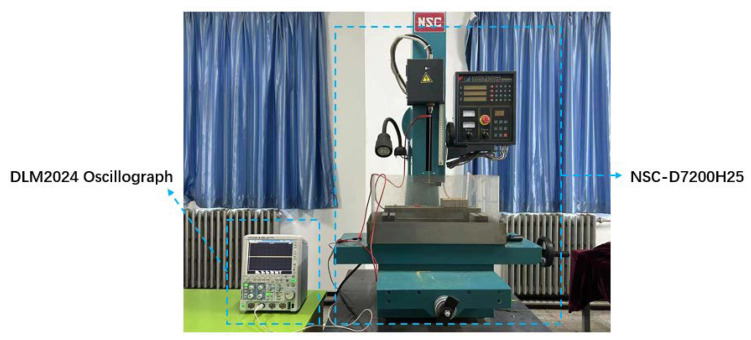
Schematic diagram of experimental equipment.

The current study involved a comprehensive series of experiments using discharge drilling equipment (NSC-D7200H25, Suzhou New Spark Machine Tool Co., Ltd., Suzhou, China). The equipment comprises a servo-feed system, a high-voltage power supply, a circuit system, a working tank, and a working fluid circulation system, enabling it to perform submerged EDM machining, as illustrated in Figure 1. During the experiments, voltage and current probes from a Yokogawa oscilloscope DLM2024, Yokogawa Electric Co., Ltd., Tokyo, Japan were affixed to the tool and workpiece to collect voltage and current waveforms throughout the machining process.

**Figure 2:**
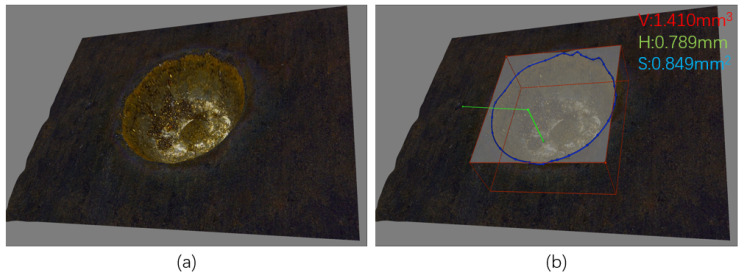
Measurements: (**a**) 3D morphology; (**b**) Specific shape parameters.

Additionally, a precision optical microscope and computer vision techniques were utilized to observe the machining results on the workpiece. Figure 2a showcases the actual 3D morphology of the machined surface captured by the microscope, while Figure 2b exhibits the morphological parameters, including machined volume, machined surface area, and machined depth, measured using accompanying computer-aided techniques. When acquiring and depicting three-dimensional outcomes, the optical microscope initially focuses on the workpiece’s surface and subsequently on the base of the machining depression. The Z-axis feed distance corresponds to the measurement from the surface to the pit’s bottom. Next, an appropriate Z-axis step size is selected for the stacking operation along the Z-axis, capturing the shape of the machining pit at each increment. Subsequently, these images are compiled, and a 3D model of the machining pit is generated. In this study, for the sake of measuring and representing the morphology of most electric discharge machining pits, we employed a systematic approach by dividing the process into 20 equidistant steps.

To ensure the adequacy of data samples for AI techniques, actual machining experiments were performed with varying input parameters. The specific variable and fixed parameters utilized in these experiments are detailed in Table 2. This comprehensive experimental setup enabled the study to gather a substantial amount of data to be employed in the development and evaluation of the proposed AI-based EDM processing result prediction model.

### 2.2. Physical Model Definition

In this study, the latest MRR prediction model proposed by Sahoo is utilized as the representative physical prediction model due to its high prediction accuracy and matching degree. Sahoo employs the ratio of the total input energy to the heat flow density of the plasma channel as the volume of the machining pit, as shown in Equation (1). By making certain assumptions, this approach effectively simplifies the complex and stochastic machining process of EDM. Consequently, it provides a systematic elucidation of the EDM machining mechanism and conducts a thorough investigation into the influence of machining input parameters on the machining results. As a result of this comprehensive research, Sahoo successfully develops a prediction model with a maximum error of 13.11%, showcasing the model’s effectiveness and accuracy in predicting EDM machining outcomes,

(1) $$V_{volumeofcrater}=\frac{Q_{powerofdischarge}}{q_{p}},$$

where $Q_{powerofdischarge}$ is the total energy input to the EDM, and $q_{p}$ is the heat flow density in the plasma channel.

The classical model developed by Sahoo incorporates a multitude of experimentally validated theories and formulas proposed by previous scholars. This includes the widely used cylindrical plasma formulation proposed by Shabgard (Equation (2)), the heat flow density formulation based on the theory of energy distribution between poles during the discharge process (Equation (3)), and the dielectric theory (Equation (4)),

(2) $$r_{p}=AI_{d}^{\alpha }t_{d}^{\beta },$$

where $r_{p}$ is the the radius of the plasma channel, $I_{d}$ is the discharge current, and $t_{d}$ is the discharge time. *A*, α and β are constants determined by experimental conditions. It depicts the relationship between the radius of the plasma channel and the discharge current as well as the discharge time.

(3) $$q_{p}=\frac{F_{A}V_{d}I_{d}}{\pi r_{p}^{2}h},$$

where $V_{d}$ is the discharge voltage, *h* is the IEG, $F_{A}$ is the inter-electrode energy distribution coefficient, which represents the portion of the total energy consumed by EDM that acts on the workpiece.

(4) $$h=\frac{V_{d}}{\varepsilon },$$

where ε is the dielectric strength. It describes the dielectric strength of a particular medium when an electrical breakdown occurs.

By bringing these equations into Equation (1), Equation (5) can be obtained:

(5) $$VMRR=\frac{\pi A^{2}V_{d}I_{d}^{2\alpha }t_{d}^{2\beta }}{F_{A}\varepsilon }.$$

Sahoo simplified the fixed and variable parameters in the formulas to suit his experimental conditions. After derivation, Sahoo’s proposed formula for predicting the volume of the machining pit from the input parameters is shown in Equation (6):

(6) $$VMRR=KV_{d}I_{d}t_{d}.$$

Here, *K* is a parameter that unifies multiple quantified influences (see Equation (7)), providing a rough reflection of the machining state. In Sahoo’s study, the value of *K* was determined through pre-experimentation and kept fixed in subsequent investigations.

(7) $$K=\frac{\pi A^{2}K_{resolid}K_{time}}{K_{\alpha }F_{A}\varepsilon }.$$

### 2.3. Physical Principles and Data-Driven Hybrid Models

The current physical models offer a fundamental understanding of the EDM process but lack detailed descriptions of certain complex phenomena. The latest Sahoo model, for instance, overlooks factors like insufficient discharge in the actual process, re-solidification after the cooling of melted material, and variations in heat transfer properties among different materials. Additionally, the use of a constant value *K* in the Sahoo model does not adequately account for the dynamic transformation between the actual processing rate and input parameters, leading to shortcomings in its prediction results, as supported by experimental evidence. The prediction error of the physical model mainly comes from the insufficient knowledge of *K*.

On the other hand, the ANN prediction model is also a data-driven approach, functioning as a black-box experiment that relies on establishing relationships between input parameters and output results. The correspondence between inputs and outputs in the ANN prediction model heavily depends on the neural network’s learning of training parameters, choosing dataset and neural network structure critical in capturing the specific contributions of input parameters to the output results. The neural network model prioritizes achieving a numerical fit, and in its specific implementation, it may amplify or reduce the contribution of individual input parameters. Consequently, the ANN model trained with a specific dataset is optimized for that particular dataset, but it may produce significant prediction errors when faced with large variations in the data set. The primary source of prediction error in data-driven models, such as ANN, stems from the model’s inaccurate understanding of the specific contribution of each input parameter to the output metrics. This discrepancy can lead to less reliable predictions, especially when encountering diverse and complex datasets.

Upon analyzing the characteristics of the physical prediction model and the data-driven model, it can be found that both have their flaws. The physical model lacks recognition of the dynamic transformation of discharge and processing states in EDM, while the data-driven model inaccurately describes the contribution of input parameters to the results. Therefore, there is a need for a new hybrid model that can acknowledge the dynamic changes in the discharge state in EDM concerning input parameters and accurately elucidate the contribution of specific input parameters to the output.

To address these limitations, this study develops a new hybrid model that combines the features of physical and data-driven models. The prediction expression of this hybrid model is determined by deforming the physical prediction equation proposed by Sahoo in a certain manner (Equation (8)).

(8) $$VMRR=K_{process}V_{d}I_{d}^{2\alpha }t_{d}^{2\beta }.$$

In the formula, $K_{process}$ additionally takes into account the insufficient discharge, the phenomenon of re-solidification, and the heat transfer properties of the workpiece material, and the specific expression is shown in Equation (9).

(9) $$K_{process}=\frac{\pi A^{2}K_{resolid}K_{time}}{K_{\alpha }F_{A}\varepsilon }.$$

Taking into account the impact of pulse parameters and measurement errors, Equation (8) is modified or deformed to derive Equation (10).

(10) $$VMRR=K_{process}V_{d}{\left(I_{d}-a\right)}^{b}{\left(\frac{T_{on}}{T_{on}+T_{off}}\right)}^{c},$$

where *a*, *b*, and *c* are variable parameters that are determined based on actual experimental conditions, while $T_{on}$ and $T_{off}$ represent the pulse width and pulse interval, respectively. These parameters play a crucial role in the modified equation (Equation (10)) and are influenced by the specific characteristics and settings of the experimental setup.

In this model, the combination of the Backpropagation Artificial Neural Network (BP-ANN) algorithm and the genetic algorithm is employed to predict the processing rate. The process coefficient $K_{process}$ in Equation (10) establishes the connection between input parameters like voltage and current pulse parameters and the process coefficient $K_{process}$ through the BP-ANN algorithm. Simultaneously, the genetic algorithm is used to search for optimal values of variable parameters *a*, *b*, and *c* on a global scale. The genetic algorithm is well known for its ability to perform global searches and adapt to various problem types. It finds wide applications in function optimization, combinatorial optimization, parameter tuning, hyperparameter search in machine learning, and other areas.

A multi-indicator BP-ANN model is developed for EDM micro-hole machining, enabling joint prediction of multiple strongly correlated shape parameters in the machining results. The BP-ANN is a specialized type of artificial neural network based on the backpropagation algorithm. Compared to general ANN, BP-ANN efficiently adjusts the network’s weights and biases during the training process to optimize its performance. This adjustment capability helps reduce the error between predicted and actual outputs. Additionally, BP-ANN employs a nonlinear activation function, enabling it to better capture nonlinear relationships in the data, making it advantageous for dealing with complex patterns and nonlinear problems. Furthermore, BP-ANN exhibits generalization ability, allowing it the application of learned patterns to unseen data and the making of accurate predictions. For a more comprehensive understanding of the BP-ANN algorithm, please refer to Haykin’s work [24].

In the industrial context, Masoud [25] successfully utilized a BP-ANN model to predict strongly correlated outputs, achieving a remarkable prediction error control of 1.5%. This exemplifies the BP-ANN model’s capability to accurately predict strongly interrelated outputs, which is an aspect not fully recognized in the EDM field. The rational application of this predictive ability can significantly contribute to the specialized EDM processing field, where higher output result prediction is required.

**Figure 3:**
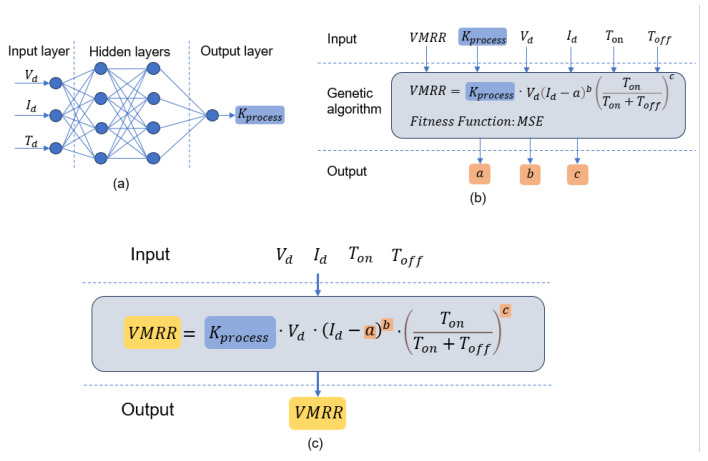
Schematic structure of the hybrid model: (**a**) the neural network training process parameters; (**b**) uses genetic algorithm to optimize the variable parameters; (**c**) the general form of the hybrid model.

Building upon prior research and accounting for the demands of EDM modeling, we introduce an innovative EDM result prediction model. This model leverages both process parameters and input voltage–current pulse parameters to anticipate machining outcomes, as depicted in Figure 3c. The prediction procedure of process coefficient $K_{process}$ is illustrated through the utilization of a BP-ANN neural network in Figure 3a, while Figure 3b exhibits the application of a genetic algorithm to optimize the optimal values of variable parameters *a*, *b*, and *c* within the model. This integration of methodologies enables a comprehensive and informed approach to EDM result prediction, addressing the intricacies of the machining process and enhancing the precision of outcome anticipation. This model incorporates a multi-output BP-ANN network for joint prediction of shape parameters such as volume, surface area, and depth in the processing results. The controlling parameters of the model include the number of hidden layers, the number of nodes in the hidden layers, the learning rate (i.e., the amount of weight update), the momentum (i.e., the amount of momentum applied during the weight update), and the number of iterations. To implement the genetic algorithm, appropriate variance functions are set based on the model characteristics, EDM processing principles, and experimental reality. The selection function further customizes the behavior of the genetic algorithm, tailoring it to the specific requirements of the EDM processing result prediction model.

## 3. Results and Discussion

### 3.1. Strongly Correlated Shape Parameters for Machining Results

The prediction of MRR in EDM is essentially equivalent to predicting the volume of the machined pit per unit time. However, in the field of EDM microporous machining, solely predicting the volume of the machined pit is insufficient, as it does not provide a comprehensive understanding of all relevant information.

During this investigation, precise measurements of macroscopic morphological parameters, including volume, pit depth, and surface area, were conducted using a precision optical microscope. Table 3 presents some of the morphological data from the test set utilized in the previous section. The data demonstrate that the pit depth and surface area exhibit variations concerning the volume, depending on the input parameters. This observation indicates that there is a substantial difference in the morphology of the EDM-machined pits under different machining conditions. The dynamic changes in the macroscopic morphology of the machining results, as observed, have not been previously highlighted in past studies. These dynamic changes can lead to distortions in the shape of the machining results, significantly impacting the performance of EDM machining.

A notable finding was that machining pits with similar shape parameters for a given metric may exhibit significant differences in other machining shape parameters. Table 4 provides a comprehensive comparison of the shape parameters of machining results under several different machining conditions, underscoring the fact that relying on a single metric shape parameter of a machining result is inadequate for assessing machining morphology. In certain cases, distinguishing between machining results with substantial morphological differences becomes extremely challenging. This highlights the complexity and intricacy of the machining process, necessitating a more comprehensive and multi-parameter approach to accurately evaluate and understand the resulting morphology.

The current literature lacks an in-depth exploration of multiple shape parameters of EDM microvia machining results. However, this study successfully identified significant correlations between shape parameters such as volume, depth, and surface area of the machined pits. To further enhance the quality of the machining results, it is essential to comprehensively understand the interconnections between these shape parameters and establish a robust relationship between the input parameters and the shape parameters of the machining results.

### 3.2. Predictive Results from Physical and Data-Driven Hybrid Models

During this investigation, 100 sets of EDM-processed data with different input parameters were utilized. To ensure a fair comparison of prediction accuracies among different models, the data sets used for building the AI model were kept consistent with those of the physical model. The test set comprised 30 groups, with 15 groups having varying pulse parameters with peak current (Group A) and 15 groups having fixed pulse parameters with varying peak current (Group B). The remaining 70 groups constituted the training set, which was randomly permuted.

In the hybrid model, the BP-ANN neural network was utilized to establish the relationship between the input parameters and the process parameters, enabling the prediction of the process parameters for a given combination of input parameters. Table 5 presents statistical measures for predicting $K_{process}$ using different neural network structures, including the coefficient of determination (R-square) and the mean absolute error (MAE) for both the training and test sets. A higher R-square value, closer to one, indicates a better fit, resulting in a smaller MAE. It is observed that all BP-ANN models have smaller prediction errors for Data set A in the test set compared to those in Data set B, suggesting that the BP-ANN models exhibit larger errors for specific data sets. Furthermore, an optimal BP-ANN network structure exists, leading to the most accurate prediction of $K_{process}$, with a mean absolute error of 2.35% for Data set A and 4.92% for Data set B.

**Figure 4:**
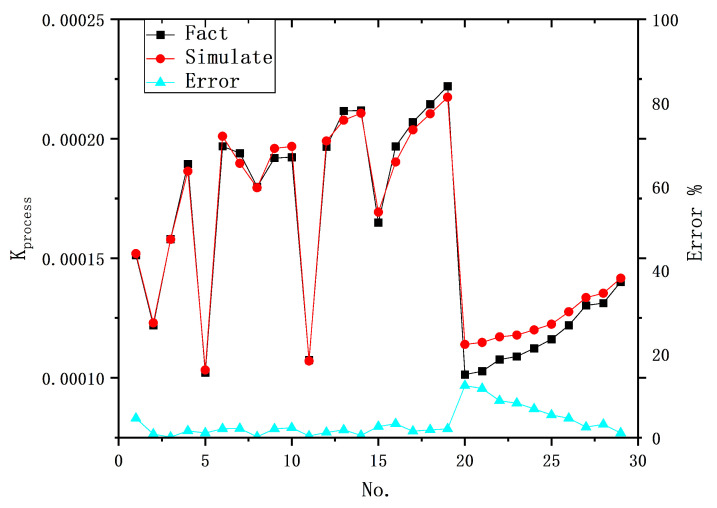
Prediction results of $K_{process}$ using 8-unit BP-ANN.

Figure 4 presents the prediction results of the eight-unit network structure compared to the original data. In the test set, the prediction results show a small error with the original data, with a maximum error of 2.913% in Group A. However, in Group B, individual experimental groups experienced a significant increase in error, with the maximum error reaching 8.45%. Notably, the twentieth experimental group exhibited the highest error, as it underwent the most substantial change in the input parameter of the discharge current, resulting in a certain increase in the error. This demonstrates that the BP-ANN model can accurately reflect the effect of the change in the discharge current law on $K_{process}$ to a certain extent.

In this study, a genetic algorithm was employed to search for the optimal values of variable parameters *a*, *b*, and *c* in the model. The population size of the genetic algorithm was set to 100, with a maximum of 1000 iterations and appropriate crossover and mutation rates. The fitness function used in the optimization process was the mean square error (MSE), which measures the accuracy of the fitting results by quantifying the difference between the fitted function and the actual data, as shown in Equation (11).

(11) $$MSE=\sum {\left(f\left(x_{i},y_{i},z_{i},a,b,c\right)-z_{i}\right)}^{2}/N,$$

where *N* is the total number of database samples.

The primary objective of the optimization process was to minimize the mean square error (MSE) to achieve the best possible fit between the output of the fitted equation and the actual data. The optimization was carried out using either the genetic algorithm, which searched for the optimal values of the variable parameters *a*, *b*, and *c* in the parameter space, resulting in a more accurate fitting equation. To determine the constraint range of the variable parameters *a*, *b*, and *c* considerations were based on established physics principles. According to the literature [26], it was found that in deionized water, the values of α and β in the plasma radius equation are approximately 0.5, which implies that the value of the variable parameters *b* and *c* should be around 1. To explore the precise values of the variable parameters “b” and “c”, their range was defined as [0.8, 1.2]. On the other hand, the variable parameter *a* represents the error in the measurement of the peak current, and its constraint range was defined as [0, 0.1], considering the accuracy of the digital oscilloscope used for measurement.

**Figure 5:**
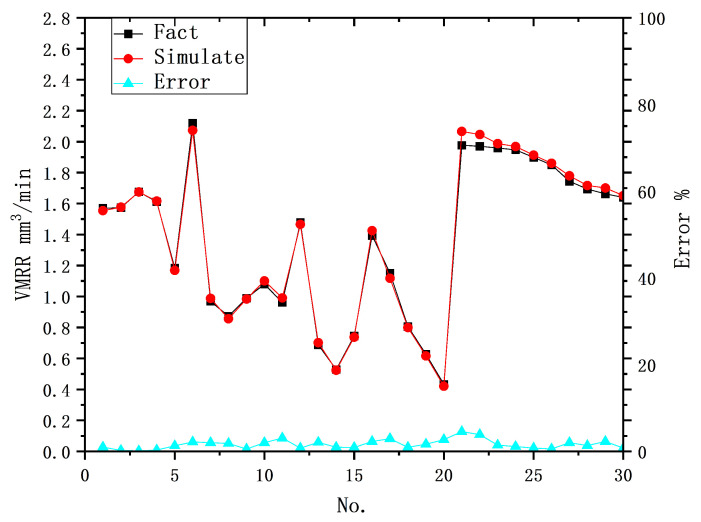
Prediction results and errors of physical principles and data-driven hybrid models for MRR.

The optimal solution found by the genetic algorithm within the given parameter range is [1.0013, 0.0043, 0.9941]. At this point, the maximum error between the predicted value and the actual experimental data is 0.0897, and MSE is 8.7916 $\times \;10^{-4}$. Figure 5 illustrates the prediction results of the hybrid model and the prediction errors. The maximum error group appears in Group B of the test set, which is 4.54%, while the maximum prediction error for Group A is 3.09%. The MSE for Group A in the test set is 3.3807 $\times \;10^{-4}$, and for Group B it is 1.4202 $\times \;10^{-3}$. The error in Group B is slightly larger than that in Group A due to the influence of Kprocess, but it still maintains high prediction accuracy. This indicates that the model has similar prediction accuracy for completely random input data and input data with specific transformation trends. The hybrid model is capable of accurately recognizing the detailed contribution of each input metric to the output results, as well as the effect of a change in a particular input metric on output results.

**Figure 6:**
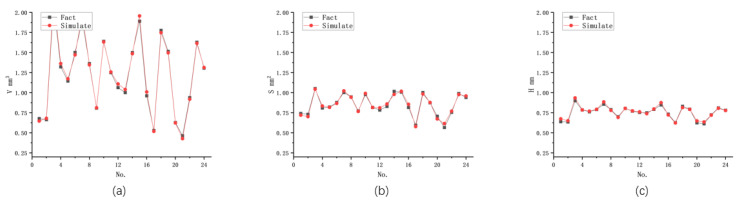
Unit 14 BP-ANN network for multimetric prediction of three strongly correlated outputs in EDM: (**a**) Volume; (**b**) Surface area; (**c**) Depth.

A multi-indicator BP-ANN algorithm was used to predict the shape parameters of three strongly correlated processing results. The prediction was based on a completely randomized dataset and different network structures, and the specific prediction results are shown in Table 6. The data revealed that the eight-unit network had a large error in predicting each indicator when strongly correlated with multiple indicators, resulting in significant prediction errors between the indicators. This indicated that the 8-unit network was not capable of accurately predicting the three strongly correlated indicators and their correlations. However, the 14-unit network showed accurate predictions for the three strongly correlated indicators, as demonstrated in Figure 6. The model accurately grasped the interrelationships between the output shape parameters and maintained good prediction accuracy. On the other hand, the 20-unit network exhibited overfitting, leading to distortion in predicting the machining volume and surface area. The findings suggest that suitable multi-indicator BP-ANN models can predict strongly correlated outputs, such as the morphology parameters of the machined pit. However, achieving high prediction accuracy requires an appropriate structure for the BP-ANN network. When predicting strongly correlated output parameters, the model needs to have enough hidden layers and neurons to capture the nuances of the correlations. A model that is too simplistic may not accurately capture the correlations, resulting in excessive prediction errors. Conversely, a model with an overly complex structure may encounter overfitting issues and increase prediction errors. In this study, the 14-unit BP-ANN model proved effective in accurately predicting the interrelationships between the output shape parameters. It demonstrated good prediction accuracy and was capable of understanding the detailed contribution of each input metric to the output results and the effects of changes in specific input metrics on the output results.

Like most data-driven models, the accuracy and robustness of the model’s predictions can be significantly impacted by the quality and completeness of the dataset used. This is especially true for ANN-based data-driven models, as they are often considered ‘black box’ models, making it challenging to discern the relationship between inputs and outputs when trained on flawed or incomplete data. On the other hand, the mathematical foundation of the hybrid model is derived from well-established theoretical research on EDM. EDM is a complex process involving various intricate physical phenomena, as discussed in the introduction. However, the academic community has not yet provided a comprehensive explanation of the entire EDM process. Therefore, it is possible that the mathematical representation of this hybrid model may not entirely capture all the nuances of the EDM process. In summary, both the quality of the training data and the mathematical structure of the hybrid model collaboratively influence the predictive accuracy and error of the model.

### 3.3. Comparison of Predictions with Traditional Physical Models and Standard Data-Driven Models

#### 3.3.1. Physical Models

The physical model proposed by Sahoo only considers the volume of the machining pit and does not account for predicting its surface area and depth. Traditionally, in classical models, the morphology of machining pits is often assumed to be either semicircular or parabolic, which oversimplifies the complex shape of machining pits and leads to prediction errors.

Before using the classical model to predict the volume of machining results, a preliminary experiment is necessary to determine the coefficient *K* in the classical model. The results of this pre-experiment are shown in Table 7. After performing calculations using Equation (6) and comparing the results with actual experimental data, the value of *K* is determined to be 2 $\times \;10^{-4}$.

**Figure 7:**
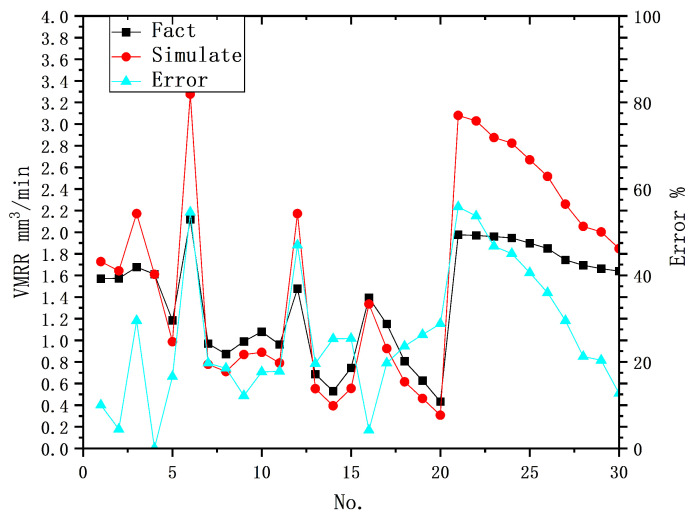
Comparison of true values with physical model predictions.

Table 8 presents the predicted MRR, actual MRR, and error values for a subset of physical models using the same test dataset. The data in the table indicate that the predictions are more accurate when they are close to the pre-experimental set. As the input parameters in the test dataset deviate further, the error gradually increases, and the maximum deviation occurs at the point of maximum change in the input parameters. This is demonstrated by under-prediction when the input parameters of the test dataset are smaller than those in the pre-experiment, and over-prediction when the input parameters of the test dataset are larger than those in the pre-experiment, as shown in Figure 7. This suggests that the physical prediction model using a fixed coefficient *K* value has a potential inherent flaw, as it cannot effectively represent the nonlinear phenomenon occurring in the actual machining process due to variations in the machining conditions, leading to deviations from the predicted results.

In real EDM processes, which are highly complex and stochastic, this instability can be caused by various factors. These factors include incomplete ionization due to impurities, resulting in shorter discharge durations [27], as well as short-circuiting of the current caused by excessive debris buildup [28,29]. Therefore, to simulate this nonlinear phenomenon using the classical model, it is essential to conduct rigorous and precise experiments to numerically quantify the influencing parameters and fully understand the effects of variations in input parameters on the discharge state. However, carrying out such experiments is time consuming and economically expensive, considering the current state of the art, and ensuring accuracy becomes challenging.

#### 3.3.2. Standard ANN Model

Table 9 presents the prediction results of ANN networks constructed from the training set data by models with various numbers of hidden layer units. The comparison of prediction results for network structures with different numbers of hidden layer neurons highlights that the number of hidden layer neurons significantly affects predictions. The ANN networks with 6 and 10 units show larger prediction errors compared to those with 8 units. This indicates that having too few or too many hidden layer neurons can lead to deviations in prediction results, which aligns with previous findings in the literature.

**Figure 8:**
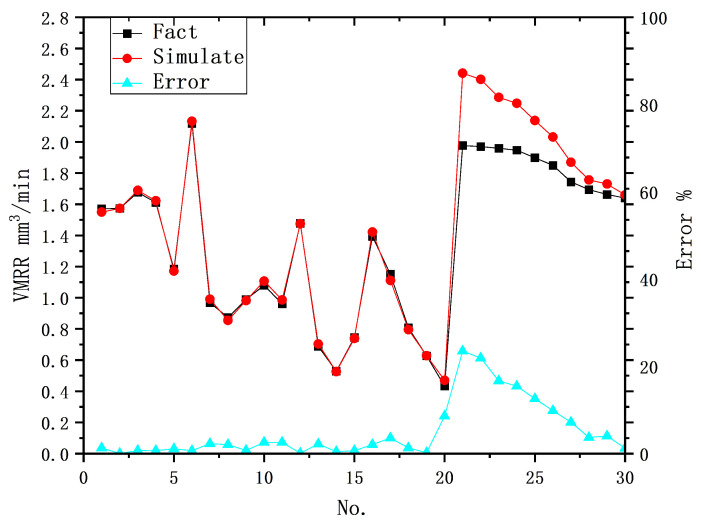
Comparison of the true values with the predicted results of the 8-unit BP-ANN model.

The prediction results of the eight-unit ANN model on the test set are shown in Figure 8. The prediction errors of the ANN model differ significantly between data from Group A and Group B. The MAE and maximum error of Group B are larger than those of Group A, suggesting that the ANN model cannot accurately express the effect of a specific change in the input data on the output results. This is because the training of the ANN model depends solely on the sample data used during training, and in the context of EDM, it does not consider the specific effects of each input parameter on the results or the interactions between the inputs. Therefore, in this study, the ANN model failed to accurately reflect the changes in output results when the peak current of the input data changed and did not precisely capture the specific contribution of the peak current to the output results.

**Figure 9:**
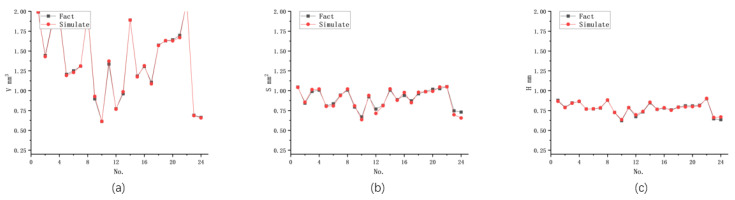
Unit 8 ANN network for multimetric prediction of three strongly correlated outputs in EDM: (**a**) Volume; (**b**) Surface area; (**c**) Depth.

The eight-unit network with the minimum error mentioned earlier was utilized to model and predict three strongly correlated shape parameters of the machining results: machining pit volume, machining pit area, and machining pit depth. The prediction results are presented in Figure 9, and the data in Table 10 show the prediction errors for each parameter.

The results indicate that the ANN model has the smallest prediction error for the machining pit volume, the second largest for the machining pit depth, and the second largest for the machining pit area. The largest prediction error is observed for the area of the machining pit. This variation in prediction accuracy is due to the fact that the specific discharge state of the EDM varies under different input parameters, leading to changes in the machining performance and affecting the machining results. As mentioned earlier, similar machining pit volumes in actual performance may correspond to numerically different machining pit areas. The standard ANN model, which predicts a single morphological parameter as an isolated indicator, does not account for the interconnections between different shape parameters, resulting in large errors when predicting strongly correlated multiple indicators. Therefore, the prediction of multiple shape parameters together using the ANN model may lead to significant deviations in results due to the complex interactions between the parameters.

#### 3.3.3. Comparison of Predicted Results

In the area of predicting MRR of processing results, a comparison between the standard ANN model and the physical model reveals that the physical model exhibits under- and over-prediction phenomena at the ends of the prediction intervals, whereas the standard ANN model demonstrates a more even and stable prediction error but fails to accurately capture the specific contributions of individual inputs.

Regarding prediction accuracy, based solely on the data from this investigation, the physical model has a mean square prediction error of 8.82%, which is greater than the 3.49% achieved by the eight-unit ANN model. The prediction accuracy of the physical model arises from its precise understanding of various states and reactions in specific processing. However, due to the multitude of complex and random physical phenomena present in the EDM process, it is not currently feasible to provide a complete and exhaustive description of all the information in EDM. As a result, the modeling process of EDM using physical models inherently lacks certain information, leading to errors in classical models. On the other hand, the learning rule of ANN involves using the rapid descent method and backpropagation to continuously adjust the weights and thresholds of the network to minimize the sum of squared errors. Therefore, for a large number of random samples, prediction results obtained using the ANN model are better than those of the classical model proposed by Sahoo.

**Figure 10:**
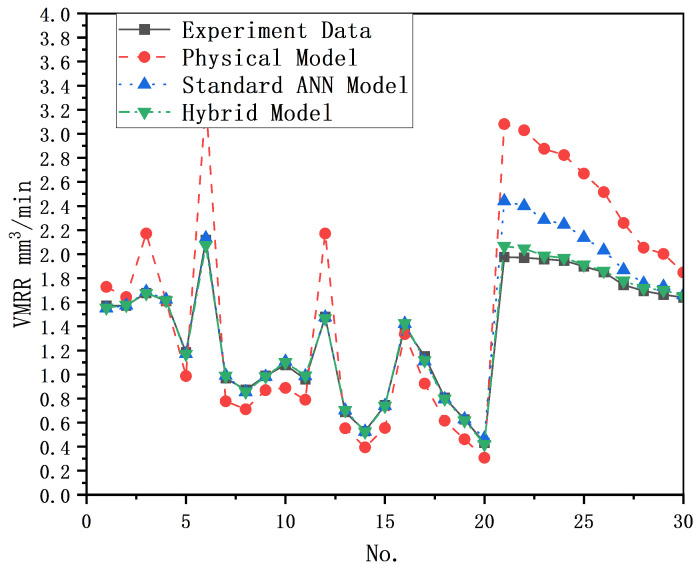
Comparison of the prediction results of different models for MRR.

In the case of single-indicator prediction targeting MRR, the standard ANN model with the best prediction results in this study exhibits a maximum prediction error of 3.49% for random samples, which is consistent with the maximum error of 2.35% of the hybrid model proposed in this study for predicting the volume of processing results. However, when predicting a specific sample with a significant change in an input parameter, the maximum prediction error and MAE of the standard ANN model are significantly larger than those of the hybrid model. This suggests that the standard ANN cannot specifically quantify the precise contribution of a particular input metric to the output results. Figure 10 illustrates the comparison of the prediction results of the developed hybrid prediction model with those of the physical and data-driven models for MRR. This demonstrates that the hybrid model proposed in this study has a remarkable advantage in capturing the specific contributions of any input parameter to the processing results. It exhibits a wider range of applicability and provides more accurate prediction accuracy compared to the physical model and the standard ANN model.

In terms of simultaneously predicting strongly correlated shape parameters such as volume, surface area, and depth of machining results, current physical models cannot predict the surface area and depth accurately. Additionally, the correlation between surface area and depth in machining results is theoretically immature and not well understood. The study compared the prediction results of three strongly correlated shape metrics using both the standard ANN model and the hybrid model. The prediction errors of the standard ANN model for volume, surface area, and depth were 3.48%, 10.21%, and 5.38%, respectively, while the hybrid model yielded lower prediction errors of 3.56%, 5.29%, and 3.98%, respectively. The hybrid model demonstrated more accurate and stable predictions, especially for the surface area and depth, compared to the standard ANN model. It effectively captured the interactions between multiple metrics and exhibited evenly distributed errors, making it superior in predicting machining results.

Table 11 provides a comprehensive comparison of the strengths and weaknesses of the physical model, the standard ANN model, and the proposed hybrid model, which combines physical principles with data-driven approaches to predict machining results in the field of EDM microvia machining. The hybrid model developed in this study is particularly effective in predicting MRR. It accurately reflects the dynamic machining phenomenon when input parameters are varied and recognizes the contribution of each input parameter to the machining results based on physics principles. The hybrid model is capable of accurately predicting both random and specific samples, displaying high prediction accuracy and even distribution of errors. Moreover, when predicting strongly correlated shape parameters, the hybrid model demonstrates evenly distributed prediction errors and can effectively capture the interaction relationship between these parameters, showcasing high prediction accuracy.

In conclusion, the proposed hybrid model shows significant improvements over the standard ANN model and current physical models, especially when dealing with strongly correlated shape parameters. Its ability to capture complex interactions between multiple metrics and its reliance on both physical principles and data-driven methods make it a promising approach for predicting machining results with enhanced accuracy and stability in the field of EDM microvia machining. This indicates that the hybrid model proposed in this study fully combines the advantages of the physical model and the data-driven model, and has a wider scope of application and more accurate prediction accuracy. The process of establishing the prediction equation quantifies the impact of specific reactions and processes in EDM on the result, which makes the prediction equation have enough theoretical support to ensure that the specific impact of each input index on the machining result is fully reflected. In the process of optimizing the variable parameters in the prediction equation, the detailed experiments in the previous literature offer a good constraint on the range of the variable parameters, which greatly accelerates the optimization process and improves the optimization results. The combination of physical and data-driven principles greatly improves the comprehensive performance of the prediction model, making it more accurate, more efficient, and more widely applicable.

## 4. Conclusions

In this study, a novel multi-parameter EDM prediction model was designed, combining physical and data-driven modeling, and its accuracy in predicting MRR and strongly correlated shape parameters was verified through extensive experiments. The research also investigated the interactions between the three morphological indicators of machining results and their dynamic transformations with the input parameters in EDM microvia machining, providing a deeper understanding of the macroscopic morphology of EDM output results. Additionally, the study compared the prediction accuracies of the physical model and the standard ANN model proposed by Sahoo for EDM processing results, along with the investigation of the classical model, the standard ANN model, and the new hybrid multi-indicator BP-ANN model. This comparison highlighted the shortcomings of the physical model and the standard ANN model while showcasing the advantages of the newly proposed hybrid model. A potential application of the high-precision prediction model proposed in this study in the industry is to serve as a model in model predictive control methods to achieve topological optimization of micro EDM processing rate and processing result quality. The main conclusions drawn from this study are as follows:

1. The relationship exists between different input parameters in EDM microvia machining and different macroscopic morphologies of machining pits. The ratios between the volume, depth, and area of the machining pits change dynamically with the machining conditions, indicating the complexity of the morphology of the machining pits. Relying solely on the machining pit volume as an evaluation index in EDM microvia machining is insufficient.
2. A hybrid model was developed that effectively combines physical principles with data-driven approaches. This model demonstrated the ability to simultaneously and accurately predict the machining rate in EDM microvia machining, as well as three strongly correlated morphological parameters of the machining results. Under the experimental conditions of this study, the model achieved an impressive maximum prediction accuracy of 4.92% for the machining rate and a maximum prediction error of 5.28% for the strongly correlated multiple metrics. These results highlight the robustness and reliability of the hybrid model in predicting various critical aspects of EDM microvia machining.
3. The limitations of both the classical model and the standard ANN model became apparent in a comparative investigation using the same experimental dataset. The classical model could only predict the processing rate of the machining results and exhibited inherent flaws in its approach. On the other hand, the standard ANN model struggled to accurately discern the individual contributions of input parameters to the processing rate, leading to significant errors when predicting strongly correlated metrics. In contrast, the newly developed hybrid model showcased remarkable capabilities. It adeptly responded to the dynamic changes observed in actual machining and accurately recognized the specific contributions of input parameters to the output indexes. Additionally, the model effectively captured the interconnections between strongly correlated outputs, demonstrating its comprehensive and accurate predictive power. This validation significantly highlights the importance and promising potential of the hybrid model. By successfully combining physical principles with data-driven methods, the hybrid model fills a crucial gap in the field of machining prediction. Its ability to overcome the limitations of traditional models and produce reliable and precise results underscores its significant contribution to the advancement of research and application in EDM microvia machining.

## Figures and Tables

**Table 1:** ANN network structure for predicting MRR used in different kinds of literature.

Author	Hidden Layer	Units	Input Parameters
Machno [19]	1	3	5
Ozkavak [22]	1, 2	5, 10	4
Ganapathy [23]	1	6	4
Mondal [20]	1	10	3

**Table 2:** Experimental variable and fixed parameters.

Parameters	Type	Level	Value
Peak current (A)	Variable	25	3∼30 (increase at intervals of 1.5)
Discharge time (µs)	Variable	2	10, 13
Interval time (µs)	Variable	3	1, 2, 5
Discharge voltage (V)	Fixed	1	25
Machining time (s)	Fixed	1	60
Dielectric fluid	Fixed	1	Distilled water
Tool	Fixed	1	Hollow brass tubes with a diameter of 1 mm
Workpiece	Fixed	1	Cast iron plate

**Table 3:** Morphological parameter results of ten different parameter combinations.

No. of Exp.	$I_{p}$ (A)	$T_{\mathrm{on}}$ (µs)	$T_{\mathrm{off}}$ (µs)	V (mm^3^)	S (mm^2^)	H (mm)	V/(S*H)
01	24.5	10	5	1.4101	0.849	0.789	0.475
02	13	10	5	1.0635	0.784	0.753	0.555
03	18	15	1	1.7610	1.003	0.826	0.470
04	24.5	15	1	1.9921	1.046	0.879	0.461
05	19.5	10	5	1.2693	0.813	0.774	0.496
06	26	10	5	1.4435	0.843	0.791	0.462
07	15	10	2	1.4632	0.960	0.791	0.519
08	24.5	10	2	1.7763	1.002	0.83	0.468
09	15	13	2	1.5219	0.990	0.794	0.517
10	30	10	5	1.5144	0.874	0.795	0.459

**Table 4:** Comparison of shape parameters of machined pits with different morphologies.

No.		V	S	H
01	A	1.5053	0.8756	0.793
	B	1.4999	1.0165	0.792
	error	0.36%	16.09%	0.13%
02	A	1.7763	1.0020	0.830
	B	1.8981	1.0024	0.856
	error	6.86%	0.03%	3.13%
03	A	1.3214	0.808	0.782
	B	1.4777	0.8701	0.784
	error	11.83%	6.21%	0.26%

**Table 5:** Predicting $K_{process}$ statistics using different network structures.

Units	R-Square	MAE in A	MAE in B
4	0.94916	5.32%	12.83%
6	0.97578	5.26%	12.89%
8	0.98303	2.91%	8.45%
10	0.97935	5.04%	10.53%
12	0.96930	7.87%	13.76%

**Table 6:** Results of different BP-ANN network structures for prediction of strongly correlated multiple indicators.

Units	Output Parameter	R-Square	MAE	Error-Max(%)
8	V	0.98085	0.0503	31.76
	S	0.90281	0.0332	17.73
	H	0.91516	0.0137	10.62
14	V	0.99422	0.0281	3.56
	S	0.96945	0.0191	5.29
	H	0.95576	0.0121	3.98
20	V	0.99414	0.0263	16.87
	S	0.93841	0.0225	14.94
	H	0.97166	0.0099	5.33

**Table 7:** Electrical parameters and processing results used in the pre experiment.

No.	$I_{d}$	$T_{\mathrm{on}}$ (µs)	$T_{\mathrm{off}}$ (µs)	V (mm^3^)
01	8	10	5	0.7676
02	8	10	2	0.9615
03	8	13	2	1.0009
04	8	15	1	1.0798

**Table 8:** Processing results of ten different parameter combinations.

No.	$I_{d}$	$T_{\mathrm{on}}$ (µs)	$T_{\mathrm{off}}$ (µs)	VMRR-Fact (mm^3^/min)	VMRR-Simulate (mm^3^/min)	Error (%)
1	17.5	10	2	1.5706	1.7281	−10.0296
5	10	10	2	1.1844	0.9875	16.6279
10	8	15	1	1.0797	0.8888	17.6923
15	5	15	1	0.7448	0.5554	25.42342
20	3	13	2	0.4329	0.3081	28.8337
25	26	13	2	1.8987	2.6702	−40.6345
30	18	13	2	1.6399	1.8486	−12.7243

**Table 9:** Prediction results and errors for different network structures.

Units	R-Square	MAE	Error (%)
4	0.99778	0.017406	4.01%
6	0.99834	0.015122	3.88%
8	0.99884	0.014636	3.49%
10	0.99762	0.016330	4.63%
12	0.98379	0.028043	12.86%

**Table 10:** Statistical data on the prediction of single indicators by 8-unit BP-ANN network.

Units	Input Parameter	R-Square	MAE	Errormax (%)
8	V	0.9988	0.01463	3.49
8	S	0.9368	0.02117	10.21
8	H	0.9781	0.00773	5.38

**Table 11:** Comparison of the applicability and reliability of different models.

Models	Prediction of MRR	Dynamics of Process	Prediction of Correlated Multi-Shape Parameters
Physical model	Significant overfitting or underfitting exists	unable to embody	Unable to predict
ANN model	Mean error distribution and high accuracy	able to embody	Uneven error distribution with large errors
Hybrid model	Mean error distribution and high accuracy	able to embody	Even error distribution, capturing interaction relationships

## Data Availability

Data are contained within the article.
